# Clinical Relevance of Liver Involvement in the Clinical Course of Systemic Sclerosis

**DOI:** 10.3390/jcm11040966

**Published:** 2022-02-12

**Authors:** Maria Lorena, Mattia Bellan, Maia Lepore, Daniele Sola, Roberta Pedrazzoli, Cristina Rigamonti, Carla De Benedittis, Giulia Francesca Manfredi, Antonio Acquaviva, Stelvio Tonello, Manuela Rizzi, Rosalba Minisini, Mario Pirisi, Pier Paolo Sainaghi

**Affiliations:** 1 Department of Translational Medicine, Università del Piemonte Orientale (UPO), 28100 Novara, Italy; 20035182@studenti.uniupo.it (M.L.); mattia.bellan@med.uniupo.it (M.B.); 20009545@studenti.uniupo.it (M.L.); cristina.rigamonti@uniupo.it (C.R.); 20013723@studenti.uniupo.it (C.D.B.); 20030060@studenti.uniupo.it (G.F.M.); 20030227@studenti.uniupo.it (A.A.); stelvio.tonello@med.uniupo.it (S.T.); manuela.rizzi@med.uniupo.it (M.R.); rosalba.minisini@med.uniupo.it (R.M.); mario.pirisi@med.uniupo.it (M.P.); 2 Internal Medicine and Rheumatology Units, AOU Maggiore della Carità, 28100 Novara, Italy; daniele.sola@maggioreosp.novara.it (D.S.); roberta.pedrazzoli@maggioreosp.novara.it (R.P.); 3 CAAD—Center for Translational Research on Autoimmune and Allergic Disease, Maggiore della Carità Hospital, 28100 Novara, Italy

**Keywords:** liver fibrosis, systemic sclerosis, transient elastography

## Abstract

Liver involvement in systemic sclerosis (SSc) is rare. We evaluated the prevalence of liver fibrosis and hepatic autoimmunity in SSc patients in a retrospective observational cohort (97 SSc or mixed connective tissue disease with sclerodermic manifestations patients undergoing transient elastography, evaluating liver stiffness (LS) and controlled attenuation parameter (CAP), due to clinical indications along with biochemistry assessments and major antibodies associated to liver autoimmunity). Among them, 11 had LS ≥ 7.5 kPa and 5 showed an LS compatible with cirrhosis (LS ≥ 12.5 kPa). Predictors of LS ≥ 7.5 fibrosis were alcohol consumption (>14 or >7 alcoholic units/week for men and women, respectively), waist circumference (>102 or >88 cm for men and women, respectively), elevated alkaline phosphatase, and anti-La and anti-mitochondrial antibody (AMA) positivity. Six patients had CAP values compatible with severe steatosis (≥280 dB/m). Waist circumference, body mass index and diabetes mellitus were significant predictors of steatosis. Out of 97 patients, 19 were positive for AMA, 4 for anti-Sp100, 1 for anti-Gp210 and 7 were diagnosed with primary biliary cholangitis. Among SSc patients, hepatic fibrosis biomarkers and AMA prevalence are relatively high, suggesting the opportunity of performing a transient elastography and a screening for hepatic autoimmunity at diagnosis and/or during disease progression.

## 1. Introduction

Systemic sclerosis (SSc) is an autoimmune connective tissue disease (CTD) characterized by fibrosis of the skin and internal organs [1]. The pathophysiology of SSc is complex and still under investigation: vascular damage in genetically susceptible individuals, elicited by environmental factors, induces the activation of endothelial cells. These cells release cytokines and adhesion molecules, triggering the immune system. As a result, activated leukocytes and macrophages produce and release pro-fibrotic cytokines that stimulate fibroblasts, eventually causing fibrosis [2,3].

Fibrotic changes of the internal organs, such as lungs, heart, kidneys, and gastrointestinal tract, characterize the clinical course of both limited (lcSSc) and diffuse cutaneous SSc (dcSSc), potentially leading to organ dysfunction [1,4]. However, liver involvement is less defined. Indeed, alterations in liver enzymes are relatively common in CTDs and potentially multifactorial: hepatotoxic drugs, viral infections, and direct liver involvement by the underlying CTDs are all potential explanations [5]. Moreover, a CTD may overlap with a liver autoimmune disease; the association with primary biliary cholangitis (PBC) is the most commonly observed among SSc patients, followed by autoimmune hepatitis (AIH) [6,7,8]. Occasionally, cases of primary sclerosing cholangitis (PSC) and nodular regenerative hyperplasia associated with SSc have also been reported [9,10,11].

Beyond biochemical alterations, there is a paucity of data about the real prevalence of liver fibrosis in SSc, which has been reported to be around 13% [12]. A deeper knowledge is therefore required to assist clinicians in decision-making, particularly to evaluate the need for liver assessment when the diagnosis of CTD is performed and for liver fibrosis monitoring during the clinical course of the disease.

This study aims to evaluate the prevalence of hepatic fibrosis in SSc patients through transient elastography. The secondary end-points were the assessment of the prevalence and type of hepatic autoimmunity; steatosis prevalence; analysis of factors associated to liver steatosis and fibrosis.

## 2. Materials and Methods

### 2.1. Study Population

An observational, retrospective study was conducted on 11,392 electronic charts of patients at the rheumatology clinic of the academic hospital “Maggiore della Carità” in Novara, Piedmont, Italy. All clinical records of patients in follow-up between January 2018 and February 2020 meeting the following criteria were reviewed:

- age > 18 years;
- SSc diagnosis according to ACR/EULAR 2013 criteria [13];
- mixed connective tissue disease (MCTD) with sclerodermic features according to Kasukawa criteria [14];
- patients who underwent hepatic elastography in the previous two years.

No exclusion criteria were applied.

We identified 225 patients with a diagnosis of SSc or MCTD; the result of a transient elastography was available for 97 of them.

Clinical history, in particular SSc signs and symptoms, comorbidities, ongoing treatments and exposure to liver disease risk factors, were derived from clinical records including anthropometric data, in particular height and weight, measured in light underwear and used to calculate the body mass index (BMI), which was considered elevated if ≥30. Waist circumference (WC) was measured as halfway between the costal edge and the crista [15,16]. In order to analyze such parameters as a categorical variable, we set up a gender-specific cut-off and considered as altered all values >102 cm in men and >88 cm in women.

A set of laboratory data was collected, including:

- Complete blood count, alanine-aminotransferase (ALT), aspartate-aminotransferase (AST), gamma-glutamyltransferase (GGT) and alkaline phosphatase (ALP), bilirubin; creatinine and estimated glomerular filtration rate (eGFR); inflammatory markers such as C-reactive protein (CRP) and erythrocyte sedimentation rate (ESR); glycated hemoglobin;
- Autoantibodies panel including antinuclear antibodies (ANA), anti-Ro60 and anti-Ro52, anti-La, anti-Scl 70, anti-centromere, anti-ribonucleoprotein (U1RNP), rheumatoid factor, anti-double strand DNA (dsDNA), anti-mitochondrial (AMA), anti-Sp100, anti-glycoprotein-210 (Gp210), anti-liver kidney microsomal type 1 (LKM1), antibody to liver cytosol (LC1) and anti-actin antibodies.

We also derived the following score, indicative of liver fibrosis degree:

- AST to platelet ratio index (APRI), which was considered as follows: APRI < 0.7 = F1; APRI 0.1–1 = F2/F3; APRI > 1 cirrhosis [17];
- AST/ALT ratio;
- The fibrosis-4 (FIB-4) score was calculated for each patient, as previously reported. FIB-4 is an algorithm based on age, AST/ALT plasma levels, and platelets count [18].

Alcoholic consumption was recorded and considered pathological if greater than 7 alcoholic units/week in women and 14 units/week in men.

### 2.2. Transient Elastography

All patients underwent transient elastography examination by FibroScan^®^ (Echosens, Paris, France), to evaluate liver stiffness (LS) and the controlled attenuation parameter (CAP). The exam was performed according to the EASL/ALEH 2015 guidelines [19].

The transient elastography examination was only considered valid if at least 10 successful measurements were obtained with a success rate was ≥ 60%, and the interquartile range-to-median ratio (IQR/median) was ≤ 30%.

LS results were interpreted as follows: LS ≥ 7.5 kPa was considered consistent with significant liver fibrosis and LS ≥ 12.5 kPa consistent with cirrhosis [20]. CAP values ≥ 248 dB/m were considered consistent with mild steatosis (S1), ≥268 dB/m with moderate steatosis (S2) and ≥280 dB/m with severe steatosis [21].

### 2.3. Statistical Analysis

Anthropometric, clinical, biochemical, and instrumental data were registered in a database and analyzed with MedCalc v.18.10.2 (MedCalc Software, Mariakerke, Belgium). For continuous variables, the measures of centrality and dispersion were medians and interquartile ranges (IQR) and the differences between groups were evaluated with the Mann–Whitney test. The Pearson χ^2^ test was used to analyze the association between categorical variables. All variables found to have at least a trend (*p*-value ≤ 0.10) suggesting an association with liver fibrosis via univariate analysis were entered into a multivariate logistic regression model with a stepwise forward and backward approach. The threshold for statistical significance was set at 0.05 (two-tailed).

## 3. Results

### Characteristics of the Population

Out of 97 patients considered, 81 were diagnosed with SSc, 7 with MCTD and 9 with an overlap syndrome. The main clinical and demographic features of the study population are detailed in Table 1. The median age was 63 years (53–73); as expected, we observed a large predominance of females (N = 86; 88.7%).

In total, 35 patients (36.1%) had gastroenteric involvement, 24 patients (24.7%) an interstitial lung disease and 12 patients (12.4%) were diagnosed with pulmonary hypertension. With respect to the ongoing treatment for the underlying CTD, 72 patients (74.2%) were on hydroxychloroquine, 12 patients (12.4%) were on methotrexate and 28 patients (28.9%) on steroids (prednisone dose ≤ 5 mg). Ten patients (10.3%) reported excess alcohol consumption, and 13 patients (13.4%) had a known liver disease.

In Table 2, we report the results of the serological testing: anti-centromere antibodies were the most represented in our population. Hepatic autoimmunity profiling showed AMA positivity in 18 patients (18.6%); anti-Sp100 in 4 patients (4.1%) and anti-Gp210 in 1 patient (1%).

When evaluating liver fibrosis, we reported a median LS of 4.6 (3.9–5.9) kPa; 16 patients (16.5%) showed values consistent with significant fibrosis, 5 of whom (5.2%) were above the threshold considered indicative of cirrhosis. Out of the 16 patients with significant fibrosis, 7 were diagnosed with PBC, 1 with PSC, 1 with AIH, 1 with nodular regenerative hyperplasia, 3 with chronic viral hepatitis and 3 with alcoholic steatohepatitis. In 8/16 (50%), the chronic liver disease was already known before performing liver elastography.

The median APRI was 0.24 (IQR: 0.189–0.34) and the median FIB-4 1.28 (IQR: 1.01–1.85). We then tested associations between predefined potentially relevant variables and an LS ≥ 7.5 kPa. The results of the univariate analysis are reported in Table S1. AMA positivity, ALP levels, WC and alcohol consumption were associated with significant liver fibrosis. We then built a multivariate analysis model including all variables with at least a trend towards statistical significance (*p* ≤ 0.10): age, GGT and ALT plasma levels, anti-La antibodies positivity, cigarette smoke and cardiac disease. As shown in Table 3, alcohol, WC, anti-La positivity, AMA positivity and ALP were independently associated with liver fibrosis; after the backward elimination, only alcohol consumption (*p* = 0.001), elevated WC (*p* = 0.002) and ALP (*p* = 0.000) values were significantly correlated with fibrosis.

We further focused our attention on liver steatosis; the median CAP was 214 dB/m (IQR: 188–245.5). In total, 16 patients showed values consistent with S1–S2 steatosis (16.5%), while in 6 patients (6.2%) severe steatosis was highly likely.

In Table S2, we report the univariate analysis for predictors of liver steatosis. In particular, diabetes (Odds Ratio = 14.33 (1.84–111.45), *p* = 0.011), elevated BMI (Odds Ratio = 11.7 (1.31–105.41), *p* = 0.028) and WC (Odds Ratio = 12.08 (1.34–108.96), *p* = 0.026) were all significantly associated with CAP values ≥ 280 dB/m. According to multivariate analysis, the presence of diabetes was the only significant predictor of liver steatosis in these patients (Table S3).

## 4. Discussion

In the present paper, we show that among SSc patients, the proportion of those with significant liver fibrosis is not negligible, but can be predicted by a history of excessive alcohol consumption, large WC and high ALP levels. These findings deserve a deeper evaluation.

According to our data, 16.5% of the study population showed significant liver fibrosis; of them, 5.2% were cirrhotic according to the results of transient elastography. Our data are in line with those obtained by Lee et al., who found a similar proportion of patients with values suggestive of fibrosis (13.6%) [12]. Although we did not compare our cohort to a control group, the observed prevalence of fibrosis is higher than expected in the general population; indeed, it has been reported to range between 5.6% and 9%, depending on the cut-off used [22,23,24]. Furthermore, in an autopsy series of 58 SSc patients and 58 matched controls, D’Angelo et al. reported that pathological findings suggestive for liver damage were significantly more prevalent in SSc patients than in controls [25]. Interestingly, only half of our patients with LS consistent with significant fibrosis were known to have chronic liver disease before performing transient elastography.

The statistically significant association with elevated alcohol consumption and waist circumference is similar to that reported for the general population [26,27,28]. Specifically looking at immune alterations, anti-La and AMA positivity were also associated with fibrosis. AMA positivity is a diagnostic criterion for PBC along with ALP elevation; thus, its association with liver fibrosis is not surprising. Anti-La antibodies are not specific for SSc and their presence in this context may be suggestive of an overlapping autoimmune liver disease; indeed, anti-La antibodies positivity has been reported in around 3% of patients with autoimmune liver diseases [29], and in up to 7% of patients affected by PBC [30]. The association with specific antibody profiles, however, was blunted by ALP, which is probably a more reliable marker of a clinically relevant seropositivity in the context of PBC.

PBC is the autoimmune liver disease most commonly overlapping with SSc. The prevalence of SSc in PBC patients is 2.8% [31]. Similarly, the prevalence of PBC in the setting of SSc has been estimated to be around 2.5% [32], and possibly even higher. Consistently, in our population, there was a high proportion of patients testing positive for PBC-associated antibodies. Among them, around 18% were AMA-positive, while a lower seropositivity was reported for anti-Sp100 and anti-Gp210 antibodies. Our findings are in line with previous reports, according to which AMA positivity among SSc patients ranges from 7 to 18% [33,34,35]. The estimated seroprevalence for AMA in the general population is much lower, ranging between 0.5% and 0.89%; this is obviously a relevant association, although AMA positivity is not diagnostic for PBC [36].

As for other markers of autoimmunity, Cavazzana et al. reported a low prevalence of anti-Sp100 (2.5%) and anti-Gp210 (0.5%) in a study cohort of SSc patients, rates that are comparable with those we observed [34].

We also evaluated the possible effects of concurrent immunosuppressive drug therapy on liver fibrosis or steatosis risk. The majority of our patients were treated with hydroxychloroquine, while only few were treated with prednisone or methotrexate as immunosuppressants. According to univariate analysis, we did not find any relevant effect of these drugs on liver fibrosis or steatosis risk, presumably due to the limited number of patients receiving them.

We also evaluated the degree of liver steatosis: the estimated prevalence of severe steatosis, defined in cases of CAP values above 280 dB/m, was around 6%, similar to what was observed in the general population [21]. Moreover, the predictors of liver steatosis are the same as can be observed in otherwise healthy subjects: diabetes, elevated BMI and elevated waist circumference [26,27].

Interestingly, even if an elevated BMI was recognized as a predictor of liver steatosis, in our SSc population the median BMI (22.89, IQR: 19.9–26.2) was comparable to that of the general Italian population, estimated in a recent work by Maffoni and coworkers (22.5, IQR: 20.3–25.2) [37].

The main limitations of our study are its retrospective nature, making it susceptible to patient allocation bias, as we performed liver transient elastography only on those patients where such clinical practice was justified by the suspicion of a liver involvement (i.e., suspected or confirmed autoimmune hepatitis, modification in cytonecrosis and cholestasis indexes, known hepatic conditions, presence of metabolic risk factors), explaining why the proportion of liver involvement in our SSc patients might have been overestimated, and the small sample size of the study population, though for the latter the rarity of the disease needs to be considered. Finally, the cross-sectional design did not allow us to draw conclusions about the variation in liver involvement over time.

In conclusion, we reported an LS consistent with significant fibrosis in around 16% of patients, which is a prevalence higher than expected in the general population. This observation suggests that rheumatologists caring for SSc patients should request a complete autoantibody profile, including markers of liver autoimmunity, at diagnosis. Moreover, the liver biochemistry profile should be carefully evaluated at diagnosis and/or along the disease course, taking into consideration performing a transient elastography. For this latter non-invasive test, priority should be given to patients with histories of excess alcohol consumption, large waist circumferences, anti-La and AMA positivity, as they are at higher risk for the development of chronic liver disease. On the other hand, the prevalence of significant steatosis is lower, and it is related to the same variables as are associated with steatosis in the general population.

## Figures and Tables

**Table 1:** Main characteristics of the study population.

Duration of disease, years	7.5 [4–15]
NYHA class 1/2/3 dyspnea	51/22/3 (52.6/22.7/3.1)
Weight, kg	59 [51–68]
Height, cm	160 [154–164]
BMI, kg/m^2^	22.89 [19.9–26.2]
Waist circumference, cm	81.5 [74–95]
Arm circumference, cm	25 [23–27]
Smoker	21 (21.7)
Arterial hypertension	47 (48.5)
Cardiovascular disease	16 (16.5)
Active cancer	13 (13.4)
Diabetes	5 (5.2)
HCQ	72 (74.2)
Steroids	28 (28.9)
MTX	12 (12.4)
Hb, g/dL	13 [12.1–13.9]
Platelets, ×10^3/^μL	244 [194.5–279.5]
eGFR (mL/min)	93 [68.5–101]
CRP, mg/L	0.15 [0.04–0.6]
ESR, mm/h	15.5 [8–25]
ALT, IU/L	18 [13.8–26.3]
AST, IU/L	23 [19–29]
AST/ALT Ratio	1.19 [0.94–1.67]
GGT, IU/L	17 [12–43.3]
ALP, IU/L	154.5 [122.5–213.5]
Bilirubin, mg/dL	0.6 [0.5–0.8]
HbA1C, mg/dL	5.4 [5.1–5.6]

Continuous variables are expressed as median [interquartile range]. Categorical variables are expressed as frequency (percentage). Abbreviations—NYHA 1: no limitations of physical activity; NYHA 2: slight limitation of physical activity; NYHA 3: marked limitation of physical activity; BMI: body mass index; HCQ: hydroxychloroquine; MTX: methotrexate; Hb: hemoglobin; eGFR: estimated glomerular filtration rate; CRP: C-reactive protein; ESR: erythrocyte sedimentation rate; ALT: alanine-aminotransferase; AST: aspartate-aminotransferase; GGT: gamma-glutamyltransferase; ALP: alkaline phosphatase; HbA1C: glycated hemoglobin.

**Table 2:** Autoantibody profile in the study population.

Autoantibody	N. Positive Patients (%)
ANA	82 (84.5)
Anti-Ro	16 (16.5)
Anti-Ro60	12 (12.4)
Anti-Ro52	9 (9.3)
Anti-La	4 (4.1)
Scl70	17 (17.5)
Anti-centromere	48 (49.5)
Anti-U1RNP	11 (11.3)
RF	15 (15.5)
Anti-dsDNA	6 (6.2)
AMA	18 (18.6)
Anti-Sp100	4 (4.1)
Anti-Gp210	1 (1)
Anti-LKM1	- (-)
Anti-LC1	- (-)
Anti-actin	- (-)

Categorical variables are expressed as frequency (percentage). Abbreviations—ANA: antinuclear antibodies; Anti-U1RNP: anti ribonucleoprotein antibodies; RF: rheumatoid factor; Anti-dsDNA: anti-double strand DNA; AMA: anti-mitochondrial antibodies; Gp210: Anti-glycoprotein-210 antibodies; LKM1: anti-liver kidney microsomal type 1 antibody; LC1: Antibody to liver cytosol.

**Table 3:** Stepwise logistic regression for liver fibrosis.

	β	Standard Error	*p*
Age	0.103	0.089	0.254
Heart disease	0.114	0.089	0.206
Smoke	0.020	0.096	0.834
**Alcohol**	**0.301**	**0.086**	**0.001**
**Waist circumference**	**0.219**	**0.088**	**0.015**
**Anti-La**	**0.175**	**0.085**	**0.044**
**AMA**	**0.203**	**0.094**	**0.034**
**ALP**	**0.478**	**0.101**	0.000
GGT	-0.163	0.113	0.155
ALT	0.177	0.092	0.059

Abbreviations—AMA: anti-mitochondrial antibodies; ALT: alanine-aminotransferase; GGT: gamma-glutamyltransferase; ALP: alkaline phosphatase. Bold text highlights the statistically significant results.

## Data Availability

Data are available upon reasonable request to be addressed to the corresponding author.

## Supplementary Materials

The following supporting information can be downloaded at: https://www.mdpi.com/article/10.3390/jcm11040966/s1, Table S1. Univariate analysis of Liver Stiffness predictors. Table S2. Univariate analysis of Liver Steatosis predictors. Table S3. Stepwise logistic regression for liver steatosis.
