# Supplementary material for: Clinical Relevance of Liver Involvement in the Clinical Course of Systemic Sclerosis
Source: J Clin Med. 2022 Feb 12;11(4):966. doi: 10.3390/jcm11040966 (PMC8879679; doi:10.3390/jcm11040966)
Supplement: Supplementary file 1 [file jcm-11-00966-s001.zip › jcm-1584968-supplementary.pdf]

Supplementary material

Table S1. Univariate analysis of Liver Stiffness predictors. ILD: interstitial lung disease; HCQ: hydroxychloroquine; MTX: methotrexate; BMI: body mass index; WC: waist circumference; AC: arm circumference; ANA: anti-nuclear antibodies; Anti-RNP: anti ribonucleoprotein antibodies; Anti-dsDNA: anti double strand DNA; AMA: anti-mitochondrial antibodies; Gp210: Anti-glycoprotein-210 antibodies; ALT: alanine-aminotransferase; AST: aspartate-aminotransferase; GGT: gamma-glutamyltransferase; ALP: alkaline phosphatase.

	**Odds Ratio**	***p***
**Male gender**	2.12 [0.49-9]	0.31
**Age**	2.89 [0.92-9.09]	0.07
**Disease duration**	0.62 [0.2-1.94]	0.40
**SSc variant**	3.13 [0.38-25.71]	0.29
**ILD**	1.48 [0.46-4.81]	0.51
**Gastrointestinal involvement**	1.47 [0.5-4.37]	0.49
**Arterial hypertension**	1.45 [0.49-4.28]	0.50
**Active cancer**	1.64 [0.4-6.77]	0.49
**Cardiac disease**	2.89 [0.84-9.93]	0.09
**Diabetes**	1.28 [0.13-12.3]	0.83
**Alcohol**	**4.17[1.02-16.97]**	**0.04**
**Smoke**	2.64 [0.83-8.4]	0.10
**HCQ**	1.05 [0.31-3.61]	0.94
**Steroids**	0.73 [0.23-2.7]	0.71
**MTX**	0.42 [0.05-3.54]	0.43
**BMI**	1.78 [0.59-5.34]	0.30
**WC**	**4.13 [1.3-13.06]**	**0.02**
**AC**	1.23 [0.13-11.99]	0.86
**ANA**	1.34 [0.27-6.6]	0.72

**Anti-Ro**	0.67 [0.14-3.30]	0.63
**Anti-Ro60**	1.01 [0.20-5.14]	0.99
**Anti-Ro52**	0.61 [0.07-5.23]	0.65
**Anti-La**	5.64 [0.73-43.43]	0.10
**Anti-RNP**	2.12 [0.49-9.0]	0.31
**Anti-dsDNA**	1.00 [0.11-9.18]	1.000
**AMA**	**4.67 [1.44-15.13]**	**0.01**
**Sp100**	0.5 [0.03-9.78]	0.65
**Gp210**	15 [0.58-385.62]	0.10
**ALT**	4.44 [0.89-22.18]	0.07
**AST**	3.72 [0.57-24.28]	0.17
**ALP**	**34.09 [3.64-319.71]**	**0.002**
**GGT**	2.87 [0.9-9.2]	0.08

Table S2. Univariate analysis of Liver Steatosis predictors. ILD: interstitial lung disease; HCQ: hydroxychloroquine; MTX: methotrexate; BMI: body mass index; WC: waist circumference; AC: arm circumference; ANA: anti-nuclear antibodies; Anti-RNP: anti ribonucleoprotein antibodies; Anti-dsDNA: anti double strand DNA; AMA: anti-mitochondrial antibodies; Gp210: Anti-glycoprotein-210 antibodies; ALT: alanine-aminotransferase; AST: aspartate-aminotransferase; GGT: gamma-glutamyltransferase; ALP: alkaline phosphatase.

	**Odds Ratio**	***p***
**Male gender**	1.90 [0.1-36.11]	0.668
**Age**	1.12 [0.21-5.85]	0.894
**Disease duration**	3.24 [0.56-18.3]	0.189
**SSc variant**	0.85 [0.09-7.92]	0.891
**ILD**	3.24 [0.61-17.26]	0.169
**Gastrointestinal involvement**	4.14 [0.72-23.92]	0.113
**Arterial hypertension**	2.24 [0.39-12.85]	0.366

**Active cancer**	1.28 [0.14-11.95]	0.827
**Cardiac disease**	0.99 [0.12-9.06]	0.991
**Diabetes**	**14.33 [1.84-111.45]**	**0.011**
**Alcohol**	0.58 [0.03-11.01]	0.713
**Smoke**	0.26 [0.01-4.83]	0.367
**HCQ**	1.85 [0.21-16.62]	0.585
**Steroids**	0.48 [0.05-4.35]	0.518
**MTX**	1.58 [0.17-14.92]	0.690
**BMI**	**11.7 [1.31-105-41]**	**0.028**
**WC**	**12.08 [1.34-108.96]**	**0.026**
**AC**	4.06 [0.36-45.36]	0.255
**ANA**	2.50 [0.13-46.80]	0.541
**Anti-Ro**	0.34 [0.02-6.30]	0.467
**Anti-Ro60**	0.48 [0.03-9.0]	0.621
**Anti-Ro52**	0.65 [0.03-12.5]	0.776
**Anti-La**	1.46 [0.07-30.21]	0.806
**Anti-RNP**	1.58 [0.17-14.92]	0.690
**Anti-dsDNA**	0.98 [0.05-19.32]	0.988
**AMA**	1.09 [0.11-10.46]	0.938
**Sp100**	5.53 [0.48-63.27]	0.169
**Gp210**	5.18 [0.19-142.58]	0.331
**ALT**	2.77 [0.28-27.63]	0.386
**AST**	4.25 [0.4-45.45]	0.231
**ALP**	0.93 [0.07-18.39]	0.961
**GGT**	0.79 [0.09-7.18]	0.833

Table S3. Stepwise logistic regression for liver steatosis. For abbreviation: BMI: body mass index

	**β**	**Standard Error**	**p**
**BMI**	0.216	0.173	0.214
**Waist circumference**	0.045	0.174	0.797

**Diabetes**	**0.264**	**0.103**	**0.012**
